# Vitamin D and HIV: shine a light

**DOI:** 10.1186/1758-2652-13-S4-O42

**Published:** 2010-11-08

**Authors:** J Currier

**Affiliations:** 1 UCLA Care Centre, 9911 W Pico Blvd, Los Angeles, USA

## 

Over the past several years there has been growing interest in the high prevalence of vitamin D deficiency in both the general population and among people living with HIV infection. Vitamin D deficiency in the setting of HIV infection is likely multi-factorial with contributions from environmental factors as well as possible associations with HIV therapy. This lecture will highlight recent data on the prevalence and possible consequences of vitamin D deficiency in the setting of HIV with a focus on the relationship between vitamin D deficiency and HIV disease progression and possible metabolic and inflammatory consequences of chronic vitamin D deficiency. Finally, the talk will attempt to shine a light on areas for further investigation.

